# One Anastomosis Gastric Bypass vs. Sleeve Gastrectomy in the Remission of Type 2 Diabetes Mellitus: A Retrospective Analysis on 3 Years of Follow-Up

**DOI:** 10.3390/jcm13030899

**Published:** 2024-02-04

**Authors:** Claudio Gambardella, Federico Maria Mongardini, Maddalena Paolicelli, Francesco Saverio Lucido, Salvatore Tolone, Luigi Brusciano, Simona Parisi, Rosetta Esposito, Francesco Iovino, Luca Nazzaro, Francesco Pizza, Ludovico Docimo

**Affiliations:** 1 Division of General, Oncological, Mini-Invasive and Obesity Surgery, University of Study of Campania “Luigi Vanvitelli”, 80131 Naples, Italy; f.mongardini@gmail.com (F.M.M.); madd.paolicelli@gmail.com (M.P.); francescosaverio.lucido@unicampania.it (F.S.L.); salvatore.tolone@unicampania.it (S.T.); luigi.brusciano@unicampania.it (L.B.); simona.parisi@unicampania.it (S.P.); rosetta.esposito@policliniconapoli.it (R.E.); francesco.iovino@unicampania.it (F.I.); luca.nazzaro@unicampania.it (L.N.); ludovico.docimo73@gmail.com (L.D.); 2 Department of Surgery, Aslnapoli2nord, Hospital “A. Rizzoli”, 80076 Naples, Italy; francesco_pizza@libero.it

**Keywords:** one anastomosis gastric bypass, sleeve gastrectomy, type 2 diabetes mellitus, bariatric procedure

## Abstract

**Background.** Obesity is a prevalent condition associated with various comorbidities, impacting mortality, fertility, and quality of life. Its relationship with type 2 diabetes mellitus (DMII) is well established, with nearly 44% prevalence. Bariatric surgery has proven crucial for treating both obesity and DMII. The comparison between surgical techniques, such as sleeve gastrectomy (SG) and one anastomosis gastric bypass (OAGB), remains controversial in terms of glycemic control efficacy. This retrospective study aimed to assess DMII remission efficacy between SG and OAGB after 36 months. **Methods.** From January 2016 to September 2020, 201 patients who underwent SG and OAGB for morbid obesity associated with DMII were accurately followed-up with for 36 months, focusing on %HbA1c, DMII remission, anthropometric results, and nutrient deficiency. **Results.** Although DMII remission did not exhibit statistical significance between the groups (82% vs. 93%, SG vs. OAGB, *p* = 0.051), OAGB demonstrated a more robust association with glycemic control (Odds Ratio 0.51) throughout the entire follow-up and yielded superior anthropometric outcomes. Notably, nutrient deficiencies, excluding cholecalciferol, iron, and riboflavin, did not show significant intergroup differences. **Conclusions.** This study contributes valuable insights into the extended-term efficacy of SG and OAGB in DMII remission. The nuanced findings underscore the multifaceted nature of metabolic outcomes, suggesting that factors beyond weight loss influence diabetes resolution. Larger comparative studies are warranted to comprehensively address this issue.

## 1. Introduction

Obesity is nowadays considered a widespread condition and one of the most investigated pathologies in modern society, particularly due to its association with several comorbidities that lead to increased mortality, infertility, and a decline in quality of life. According to the World Health Organization (WHO), the relationship between obesity and type 2 diabetes mellitus (DMII) is well established, reaching almost 44%, higher than that in ischemic heart disease (23%) and certain types of cancers (7–41%) [1,2,3]. Conversely, about 90% of patients affected by type 2 diabetes are obese [4], and the prevalence of obesity-related diabetes is expected to reach 300 million by 2025 [5]. The term “Diabesity” has been coined for most patients with DMII who are overweight or obese, indicating a close link [6,7]. It has been extensively demonstrated that bariatric surgery treatment is crucial for both obesity and DMII, mainly due to its long-term efficacy [8]. In the results of the Swedish Obesity Study (SOS), the bariatric surgery group showed a 72.4% achievement of diabetes remission at 2 years, compared to the 16.4% in the control group [9]. The second Diabetes Surgery Summit (DSS-II) included bariatric/metabolic surgery in global guidelines as glucose-lowering treatments for patients affected by DMII and obesity [10]. According to the Italian bariatric surgery guidelines, a bariatric procedure is indicated for individuals with a body mass index (BMI) over 40 kg/m^2^, or a BMI > 35 kg/m^2^ combined with several comorbidities, with a focus on DMII [11]. The regression of diabetes after bariatric surgery can be attributed to both weight loss-dependent and weight loss-independent mechanisms. While malabsorptive and mixed procedures induce faster weight loss compared to restrictive ones, they also reflect a mode of metabolic adaptation in the long term [12]. Among the metabolic weight loss-independent mechanisms, improved liver insulin sensitivity, decreased liver fat content, and higher circulating free bile acids are implicated in liver gluconeogenesis and glucose hemostasis [13,14,15]. Excess adipose tissue leads to the downregulation of circulating bile acids, particularly valine and leucine, which are involved in glucose homeostasis. Weight loss is inversely correlated with circulating levels of ghrelin, which also plays a positive role in glucose homeostasis. Also, incretins are hormones that play a crucial role in the regulation of blood glucose levels, and their role is particularly significant in the context of DMII. The two main incretins involved in glucose homeostasis are glucagon-like peptide-1 (GLP-1) and glucose-dependent insulinotropic polypeptide (GIP). Individuals with type 2 diabetes often exhibit a diminished incretin effect, meaning their insulin response to oral glucose is impaired compared to non-diabetic individuals. Therefore, in the selection of the best surgical option, hormonal pathways should also be considered. Among the mixed procedures, one anastomosis gastric bypass (OAGB) is now the third most common procedure worldwide, performed after sleeve gastrectomy (SG) and Roux-en-Y gastric bypass (RYGB) [16]. A SM-BOSS randomized clinical trial found no statistically significant difference in glycemic control between sleeve gastrectomy and RYGB [17]. The aim of the current retrospective analysis is to investigate the efficacy of diabetes remission between sleeve gastrectomy (SG) and OAGB after 36 months from surgery.

## 2. Methods

### 2.1. Study Design

This study is reported according to the STROBE statement for cohort studies [18]. A retrospective monocentric study was conducted to compare the efficacy of SG vs. OAGB in the remission of DMII, in patients affected by obesity and diabetes. It was conducted according to the ethical principles stated in the Declaration of Helsinki. Written informed consent was obtained from all patients.

### 2.2. Study Setting and Study Population

From 1 January 2016 to 30 September 2020, all patients who underwent sleeve gastrectomy (SG) and one anastomosis gastric bypass (OAGB) at the Division of General Surgery of a Teaching Hospital were included in the study. Inclusion criteria were age ≥16 years, BMI over 35 kg/m^2^ according to the Società Italiana di Chirurgia (SICOB) guidelines, and type 2 diabetes mellitus (DMII) with at least one antidiabetic agent [11]. Exclusion criteria were patients who underwent revisional bariatric surgery, those affected by type I diabetes, the concomitant presence of neoplasms, psychiatric diseases, or alcoholic dependence, drug addiction, pregnancy, and inability to adequately participate in the follow-up program.

All patients underwent a routine preoperative clinical and instrumental diagnostic assessment, including anamnestic data collection, blood exams, ECG, cardiologic, anesthesiologic, and psychiatric evaluations, thoracic X-ray, pneumologist evaluation with spirometry, upper endoscopy (EGDS), complete abdomen and thyroid ultrasound, lower limb Doppler ultrasound, and nutritionist counseling. After the referral for surgery, each patient received a detailed explanation of the procedure from the medical staff and had to sign a personalized informed consent form. All operations were performed by the same experienced surgeons with over 250 bariatric procedures. Clinical data were collected in an electronic database and retrospectively analyzed.

Patients who underwent sleeve gastrectomy were assigned to Group A, while those who underwent one anastomosis gastric bypass were assigned to Group B. Both groups’ data were compared pre- and post-operatively regarding diabetes outcomes in terms of HbA1c% and antidiabetic agents.

### 2.3. Surgical Technique 

#### 2.3.1. One Anastomosis Gastric Bypass

Pneumoperitoneum was established using a standard Visiport technique (Medtronic Inc., Dublin, Ireland) at the Palmer site, with an optical insertion of a 12 mm supraumbilical port (10–15 cm from the umbilicus). Two additional 15 mm ports and one 12 mm port were placed in the upper abdomen, respectively in the left and right hypochondrium, as working ports. A subxiphoid track was created using a 5 mm port for liver retractor placement. A long, narrow gastric pouch was designed starting from beyond the crow’s foot to just lateral to the angle of His over a 36 Fr orogastric tube using a 60 mm Echelon flex^®^ (Ethicon Inc., Raritan, NJ, USA). When a hiatal hernia was present, a posterior hiatoplasty was performed using two or three non-absorbable stitches. The total count of the bowel length was not routinely performed. Gastrojejunostomy was then performed between 180 cm distally to the ligament of Treitz using a 60 mm Echelon flex^®^ (Ethicon Inc., Raritan, NJ, USA) linear stapler followed by stapler entry closure, as reported in our previous experiences [19,20]. There was no difference in the fashion of the gastrojejunostomy among the patients. An endoscopic leak and patency test were performed at the end of the procedure. One drain was positioned in the splenic site. Patients were advised to take lansoprazole 30 mg daily for 6 months. They were further recommended to take a multivitamin/mineral tablet (Bariatrifast^®^, Bio Italia S.r.l., Rome, Italy) and receive vitamin B12 injections every three months for the rest of their life.

#### 2.3.2. Sleeve Gastrectomy

The procedure began with a dissection of the greater omentum perpendicularly to the incisura angularis alongside the greater gastric curvature. Starting from this point, the greater curvature was divided upward, and the dissection was concluded once the fundus was entirely detached. Gastric transection began 5–6 cm from the pylorus and was extended until 2 cm from the angle of His over a 36 Fr orogastric tube using a 60 mm Echelon flex^®^ (Ethicon Inc., Raritan, NJ, USA). Attention was given to achieving a regular shape and avoiding an excessive narrowing of the gastric lumen at the incisura angularis while ensuring the complete removal of the posterior fundus. The stapler line was routinely reinforced using an oversewing running suture. Intraoperative endoscopy double-checked intraluminal bleeding and assessed the size and integrity of the stapler line. Patients were advised to take lansoprazole 30 mg daily for 6 months and a multivitamin/mineral tablet (Bariatrifast ^®^, Bio Italia S.r.l., Rome, Italy) for almost 6 months.

### 2.4. Outcome Measures

The postoperative follow-up comprised several appointments for clinical evaluation. Specifically, from their initial appointment, anthropometric parameters were recorded, including BMI (in kg/m^2^), mean weight (in kg), the percentage of excess weight loss (%EWL), and the percentage of total weight loss (%TWL) from the preoperative baseline. These evaluations were performed at 6, 12, 24, and 36 months. Additionally, blood investigations for nutritional status (vitamin D3, iron and ferritin, B12 vitamin, total protein, hypoalbuminemia) and %HbA1c were conducted. DMII’ remission was defined by the presence of an HbA1c < 6.0% for at least 1 year without anti-diabetes medications [21]. Oral interviews were also conducted during the clinical outpatient assessments to evaluate the number of oral antidiabetic agents taken. 

Consistent with general management, all postoperative tests and procedures were provided free of charge. Follow-ups were completed for all patients in September 2023 for subsequent data analysis.

### 2.5. Study Outcomes

The primary outcome was the remission of DMII at 36 months of follow-up in patients who underwent SG and OAGB. The secondary outcome was the evaluation of anthropometric features and nutrient deficiencies in patients who underwent SG and OAGB after 36 months of follow-up.

### 2.6. Statistical Analysis

The population was divided into 2 groups, patients who underwent SG and patients who underwent OAGB. Data were described according to each variable type. Continuous variables were expressed as the mean with its standard deviation (SD). Frequencies were used for categorical variables. *p*-values below 0.05 were considered significant. Stata 16 (StataCorp, College Station, TX, USA) was utilized for all statistical analyses. To evaluate the association between DMII’ remission with the surgical procedure, Odds Ratio (OR) analysis was performed at the maximum follow-up.

## 3. Results 

### 3.1. Study Population

From 1 January 2016 to 30 September 2020, of the 469 patients referred for severe obesity, 416 met the SICOB criteria and received bariatric surgery treatment, while DMII was present in 201 cases. One hundred and thirty-seven patients underwent laparoscopic SG (Group A), while 64 received OAGB (Group B). In the considered population of the 2011 patients included in the study, 111 were males (55.2%) and 90 were females (44.8%), with a mean age of 39.3 ± 5.3 years and a mean BMI of 46.8 ± 3.7 kg/m^2^, excess body weight (EBW) 67.4 ± 20.9 kg. Baseline demographic and pathological findings are detailed in Table 1. The mean follow-up was 39.4 ± 2.2 months.

**Figure 1:**
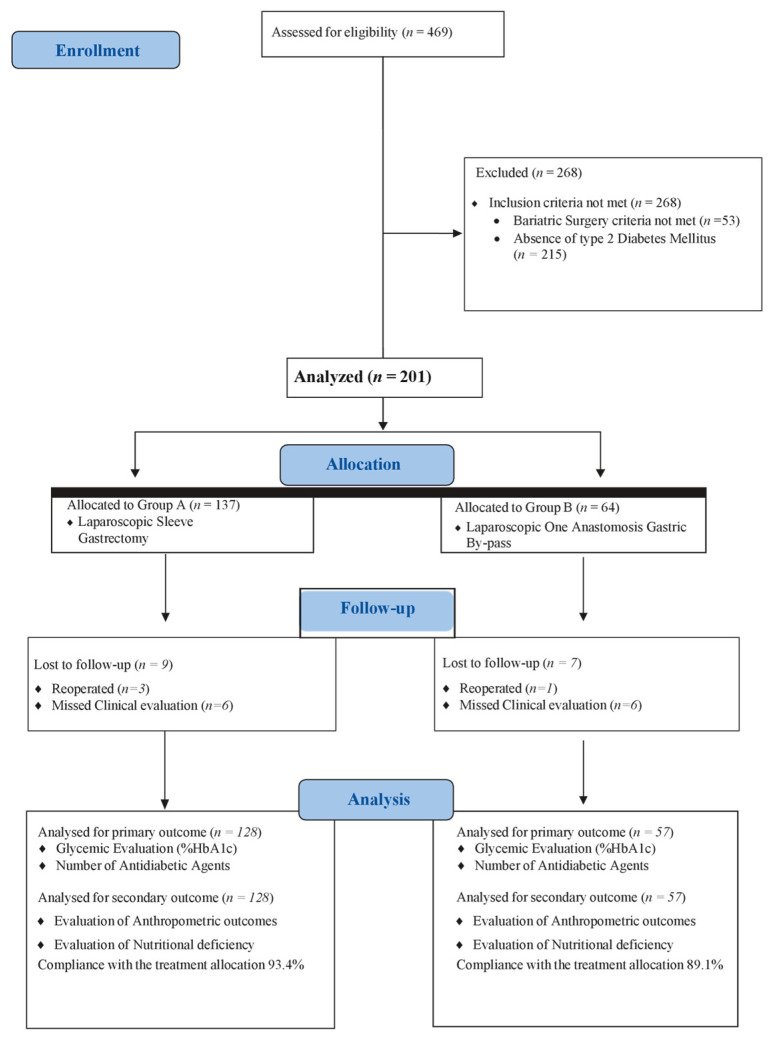
Study flowchart.

By the follow-up, 9 patients (6.6%) in Group A and 7 patients (10.9%) in Group B were excluded from the analysis. In detail, in Group A, 3 (2.2%) underwent a further abdominal surgical procedure, and 6 (4.4%) missed the follow-up. In Group B, 1 (1.6%) underwent a further abdominal surgical procedure, and 6 (9.3%) missed the follow-up (Figure 1).

### 3.2. Primary Outcome 

**Figure 2:**
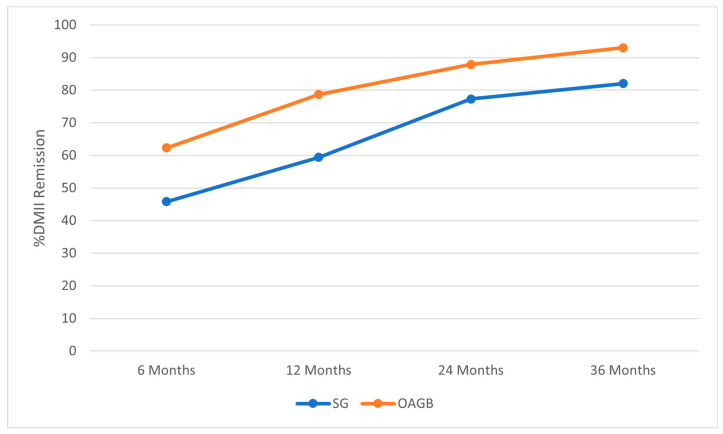
Type 2 diabetes mellitus (DMII) trend in patients who underwent SG and OAGB during the entire follow-up period.

DMII’ features at 6, 12, 24, and 36 months postoperatively are recorded in Table 2. Postoperative %Hb1Ac was 7.2 ± 1.3 in Group A and 6.9 ± 1.5 in Group B (*p* = 0.023) at 6 months, 6.5 ± 1.4 in Group A and 6.1 ± 1.6 in Group B (*p* = 0.016) at 12 months, 6.2 ± 1.3 in Group A and 5.9 ± 1.6 in Group B (*p* = 0.354) at 24 months, and 5.9 ± 1.2 in Group A and 5.7 ± 1.4 in Group B (*p* = 0.421) at 36 months. Regarding the antidiabetic agents, the numbers assumed in Groups A and B at the 36-month follow-up were 0.9 ± 0.3 and 0.6 ± 0.2, respectively (*p* = 0.059). In detail, patients who no longer used antidiabetic drugs by follow-up for the remission of DMII were 61 in Group A and 38 in Group B (*p* = 0.033) at 6 months, 79 in Group A and 48 in Group B (*p* = 0.007) at 12 months, 99 in Group A and 51 in Group B (*p* = 0.090) at 24 months, and 105 in Group A and 53 in Group B (*p* = 0.051) at 36 months (Figure 2). The Odds Ratio for evaluating the association between DMII’ remission and the surgical procedure resulted in 0.51 (0.27–0.96, *p* = 0.03), in favor of OAGB. 

### 3.3. Secondary Outcome

Anthropometric parameters at 36 months postoperatively are depicted in Table 3 and were all significantly improved in Group B. In detail, the mean weight was significatively lower in Group B (88.3 ± 8.4 kg vs. 83.5 ± 6.2 kg; *p* = 0.023), and the mean BMI was also lower in Group B (31.9 ± 5.3 vs. 29.2 ± 6.9; *p* = 0.016). Therefore, the mean %EWL (74.3 ± 13.8 vs. 83.6 ± 18.1, *p* = 0.003) and %TWL (37.9% ± 14.5 vs. 41.57 ± 12.8, *p* = 0.002) were significantly higher in Group B.

The incidence of nutrient deficiency at 36 months postoperatively is reported in Table 4, with cholecalciferol (Vit. D3) (3.9% vs. 12.3%, *p* = 0.03), iron (9.3% vs. 15.7%, *p* < 0.001), and riboflavin (Vit. B2) (2.3% vs. 8.7%, *p* = 0.043) showing significant differences. For other parameters, no statistical differences were reported.

## 4. Discussion

In the current study, we compared SG and OAGB in terms of their efficacy in diabetes remission after a 36-month follow-up. The analysis aimed to assess potential connections between weight loss, diabetes remission, and their respective time trends. 

Established metabolic surgical techniques, including RYGB and SG, have proven effective in ameliorating metabolic disorders and achieving substantial weight loss. Nevertheless, the choice between these procedures remains controversial, as the comparative efficacy in glycemic control is currently a subject of debate. Mingrone et al. [22] previously demonstrated the superiority of metabolic surgery (RYGB or biliopancreatic diversion) over conventional medical therapy for the long-term control of DMII. However, the mentioned bariatric procedure (i.e., biliopancreatic diversion) is considered, nowadays, obsolete, with sleeve gastrectomy (SG) being the most frequent and preferred choice due to its safety and lower risks [23,24]. 

Despite having similar surgical risks as RYGB, OAGB was recently considered by the International Federation for the Surgery of Obesity and Metabolic Disorders (IFSO) as the main bariatric procedure in terms of weight loss and metabolic advantages [25,26]. However, it should be considered a relatively “novel” procedure in the literature, and only few studies have analyzed the glycemic trends after OAGB compared to those of restrictive procedures (i.e., SG). Musella et al. [27] conducted a study comparing 206 patients who underwent OAGB and SG to evaluate the efficacy of these bariatric surgeries in DMII remission in morbidly obese patients after a 1-year follow-up. They found a significantly higher DMII remission rate in OAGB patients compared to SG patients. No correlation was found between the percentage change vs. baseline HbA1c and BMI reduction for both procedures (ΔHbA1c 0.4 for OAGB; ΔHbA1c 0.1 for SG). Vrakopoulou et al.’s [28] data also showed a greater remission of DMII in the OAGB group compared to SG, with 88% of patients off antidiabetic agents 3 years after surgery, reflecting their status of normoglycemia, in comparison to a surprising low 35.7% in the SG group. An interesting metanalysis by Ding et al. on five randomized controlled trials confirmed the abovementioned results, reporting that the DMII remission of T2DM in the OAGB group was more efficient at 1 year and 5 years along with a lower BMI at 5 years than the SG group [29].

To the best of our knowledge the current study is the first analyzing the DMII trends after SG and OAGB along with the anthropometric results and the nutrient deficiency at a 36-month follow-up. Our results confirm a better response for the OAGB group in terms of HbA1c changes at the 6- and 12-month follow-up. However, a trend reversal occurred during the extended follow-up; the differences between the SG and OAGB groups in terms of %HbA1c, number of antidiabetic agents (ADAs), and diabetes remission were no longer statistically significant at 24 and 36 months. The DMII remission with patients off antidiabetic agents occurred in 82% of patients in the SG group and 93% of those in the OAGB group (*p* = 0.059). However, the Odds Ratio, considering the entire follow-up length, attested the superiority of OAGB in terms of DMII’ remission (0.51; 0.27–0.96, *p* = 0.03). Notably, both procedures seemed effective in the present cohort with a long-standing history of DMII (64.9 ± 50.3 months vs. 66.1 ± 43.3 months in the SG and OAGB groups, respectively, *p* = 0.312). However, in the current retrospective study, it is hard to detail the pancreas functionality and draw conclusions regarding patients with a deteriorated pancreas. Also, Shivakumar et al., in their randomized controlled trial on medium-term results of OAGB vs. SG, reported similar diabetes remission rates in OAGB and SG patients 3 years after surgery (89.13% of OAGB patients and 81.82% of LSG patients) without reaching a statistically significant difference [30]. Conversely, Kular et al. found better metabolic strength in the omega gastric bypass/OAGB group. A DMII resolution of 92% was observed in the MGB group and 81% in the LSG group and a hyperlipidemia remission of 90% in the MGB group and 72% in the LSG group, after 5 years of follow-up [31]. 

Regarding the anthropometric outcomes, in terms of the %EWL, our study is consistent with previous studies reporting results in favor of OAGB [28,29]. The OAGB group, in fact, achieved a significantly higher %EWL of 83.6 ± 18.1%, whereas the SG group reached 74.3 ± 13.8% at 36 months postoperatively (*p* = 0.03). However, the current findings between the procedures were more similar, compared to the ones of Vrakopoulou et al. [28], who reported a significantly higher percentage of excess weight loss (%EWL) for OAGB (98 ± 29.0%) compared to SG (79.7 ± 14.5%) at 36 months postoperatively. 

Considering the inconsistent results reported in the current analysis, with excellent anthropometric outcomes after OAGB and comparable glycemic control between the two procedures, it is conceivable to argue that weight loss alone may not be sufficient to induce metabolic control. Several other factors, as yet unidentified, that influence the glycemic trend should be investigated.

Regarding nutrient deficiencies, Musella et al. [32] reported early postoperative values of vitamin D comparable between the SG and gastric bypass (OAGB and RYBP) groups at 6 months, with no significant difference between the groups. The authors, in particular, focused on the importance of regular follow-up with correct supplementation in patients undergoing OAGB to prevent nutrient deficiency. In a previous series by Pizza et al., regarding the anthropometric results and the vitamin deficiencies after OAGB performed with different biliopancreatic limb lengths (150 cm, 180 cm, 200 cm), the authors concluded that a limb length of 150–180 cm was safe and effective in terms of %EWL and comorbidity improvement with minimizing malnutrition effects even with a BMI > 50 [19]. Therefore, in order to achieve an excellent weight loss and comorbidity remission guarantying a low rate of vitamin deficiencies, in the current series, we opted for a biliopancreatic limb of 180 cm. Maria-Jose Castro et al. [33] compared SG, RYGB, and OAGB results, finding significant low iron blood levels only in the SG group at 5 years of follow-up, with no statistically significant differences in other nutritional deficiencies. In our study, considering only SG and OAGB, there were no statistically significant differences in nutrient deficiencies between the groups, except for iron deficiency, riboflavin deficiency, and vitamin D deficiency at 36 months of follow-up. 

In the current series, OAGB appeared to achieve an excellent anthropometric goal but exhibited similar glycemic control to SG at 36 months. However, despite these outcomes, and with a lower Odds Ratio in association with DMII, OAGB likely presents more serious drawbacks that should not be neglected. It is a more complex and technically demanding procedure than SG, and it may have higher rates of complications, such as leakage and nutrient deficiency. OAGB also necessitates lifelong supplementation and monitoring of vitamins and minerals, such as iron, calcium, vitamin B12, and vitamin D, to prevent anemia, osteoporosis, and neurological disorders [16,25]. Therefore, the decision between SG and OAGB should be made after a careful evaluation of the benefits and risks of each technique, as well as the patient’s preferences and expectations. Arguably, SG, considering its diffusion, reproducibility, and procedural ease, could be viewed as a safe initial approach in obese patients with diabetes, reserving the possibility of a second surgical look with OAGB in case of failure. A multidisciplinary approach, involving surgeons, endocrinologists, nutritionists, psychologists, and other specialists, is recommended to provide optimal care and follow-up. 

The current study has certain limitations in its retrospective design, medium follow-up, and limited cohort. Nevertheless, despite the relatively modest patient count in this retrospective analysis, the uniformity in preoperative characteristics and the high rate of follow-up, encompassing a complete dataset for each patient throughout the 36 months of follow-up, bestow credibility upon our study. 

## 5. Conclusions

After the previous results in favor of the malabsorptive procedure, our study provides insights into the comparative effectiveness of SG and OAGB in diabetes remission at 36 months of follow-up. However, the association between DMII remission appeared stronger with OAGB considering the entire follow-up length (Odds Ratio 0.51). The results of the primary outcome were not in accordance with the %EWL and BMI rates, which resulted to be significantly improved in the OAGB group at 36 months of follow-up. Arguably, SG, considering its diffusion, reproducibility, and procedural ease, could be viewed as a safe initial approach in obese patients with diabetes, reserving the possibility of a second surgical look with OAGB in case of failure. This indicates that weight loss alone cannot ensure the metabolic goal and that several other factors should be considered and investigated. Further, larger comparative studies are needed to address this issue.

## Figures and Tables

**Table 1:** Obese DMII patients’ demographics and preoperative anthropometric value characteristics.

	Group A (SG) 137 pts	Group B (OAGB) 64 pts	*p*
Gender (Male/Female)	76/61 (55.5%/44.5%)	35/29 (55%/45%)	0.196 **
Age (Years) °	38.9 ± 4.6	40.1 ± 5.1	0.518 *
ASA I–II/III–IV	58/79 (42.4–57.6%)	24/40 (37.5–62.5%)	0.515 **
BMI (kg/m^2^) °	46.2 ± 3.6	47.1 ± 2.3	0.438 *
Weight (kg) °	135.6 ± 20.7	137.1 ± 21.4	0.078 *
EBW (kg) °	66.5 ± 21.3	68.1 ± 28.7	0.364 *
Hypertension	59 (43.1%)	31 (48.4%)	0.475 **
Dyslipidemia	79 (57.6%)	38 (59.7%)	0.818 **
Chronic obstructive pulmonary disease	21 (15.3%)	11 (17.2%)	0.737 **
Cerebrovascular Disease	10 (7.4%)	5 (8%)	0.897 **
Smoking	59 (43.1%)	26 (41.3%)	0.744 **
Antiplatelets/Anticoagulation	19 (13.9%)	9 (13.7%)	0.970 **
Coronary artery disease	24 (17.5%)	12 (19.5%)	0.832 **
DMII-positive familial history	92 (67.1%)	41 (64.2%)	0.519 **
Age at diagnosis (Years) °	40.2 ± 6.7	41.4 ± 7.1	0.478 *
Diabetes history (Months) °	64.9 ± 50.3	66.1 ± 43.3	0.312 *
IDDM	34 (24.6%)	12 (19.6%)	0.340 **
1 Antidiabetic agent	87 (63.5%)	36 (56.2%)	0.325 **
2 Antidiabetic agents	39 (28.4%)	20 (31.3%)	0.686 **
3 Antidiabetic agents	11 (8.1%)	8 (12.5%)	0.312 **
Preoperative %Hb1AC °	7.7 ± 1.6	7.9 ± 1.8	0.163 *

Values are expressed as the number of cases or mean ± standard deviation °. DMII—type 2 diabetes mellitus, SG—sleeve gastrectomy, OAGB—one anastomosis gastric bypass, EBW—excess body weight, BMI—body mass index, IDDM—insulin-dependent diabetes mellitus, %HbA1c—percentage of glycated hemoglobin. * Unpaired *t*-test, ** Fisher’s exact test.

**Table 2:** Postoperative diabetes index at 6, 12, 24, and 36 months in patients who underwent sleeve gastrectomy and one anastomosis gastric bypass.

	Group A 133 pts	Group B 61 pts	*p*	GroupA 131 pts	Group B 60 pts	*p*	Group A 128 pts	Group B 58 pts	*p*	Group A 128 pts	Group B 57 pts	*p*
	6 Months			12 Months			24 Months			36 Months		
Number of ADAs °	1.3 ± 0.5	1.1 ± 0.3	0.012 *	1.1 ± 0.5	0.9 ± 0.3	0.012 *	0.8 ± 0.5	0.6 ± 0.2	0.064 *	0.9 ± 0.3	0.6 ± 0.2	0.059 *
Diabetes’ Remission	61 (45.8%)	38 (62.3%)	0.033 **	79 (59.4%)	48 (78.7%)	0.007 **	99 (77.3%)	51 (87.9%)	0.090 **	105 (82%)	53 (93%)	0.051 **
1 ADA	43	11	0.039 **	41	8	0.008 **	21	4	0.078 **	18 (14%)	3 (5.3%)	0.081 **
2 ADAs	27	9	0.356 **	10	3	0.502 **	7	3	0.933 **	4 (3.2%)	1 (1.7%)	0.595 **
3 ADAs	2	3	0.163 **	1	1	0.569 **	1	-	0.499 **	1 (0.8%)	-	0.503 **
%Hb1AC °	7.2 ± 1.3	6.9 ± 1.5	0.023 *	6.5 ± 1.4	6.1 ± 1.6	0.016 *	6.2 ± 1.3	5.9 ± 1.6	0.354 *	5.9 ± 1.2	5.7 ± 1.4	0.421 *

Values are expressed as the number of cases or mean ± standard deviation °. %HbA1c (%)—percentage of glycated haemoglobin, ADA—antidiabetic agent. * Unpaired *t*-test, ** Fisher’s exact test.

**Table 3:** Anthropometric outcomes after 36 months in patients who underwent sleeve gastrectomy and one anastomosis gastric bypass.

	Group A (SG) 128 pts	Group B (OAGB) 57 pts	*p*
Weight (kg)	88.3 ± 8.4	83.5 ± 6.2	0.023 *
BMI (kg/m^2^)	31.9 ± 5.3	29.2 ± 6.9	0.016 *
%EWL	74.3 ± 13.8	83.6 ± 18.1	0.003 *
%TWL	37.9 ± 14.5	41.57 ± 12.8	0.002 *

Values are expressed as mean ± standard deviation. BMI—body mass index, %EWL—percentage of excess weight loss, %TWL—percentage of total weight loss. * Unpaired *t*-test.

**Table 4:** Frequency of nutrient deficiencies after 36 months in patients who underwent sleeve gastrectomy and one anastomosis gastric bypass.

	Group A (SG) 128 pts	Group B (OAGB) 57 pts	*p*
Cholecalciferol (Vit. D3)	5 (3.9%)	7 (12.3%)	0.03 **
Iron	12 (9.3%)	9 (15.7%)	<0.001 **
Cyanocobalamin (Vit. B12)	4 (3.1%)	4 (7.0%)	0.229 **
Total Protein	6 (4.7%)	5 (8.7%)	0.278 **
Hypoalbuminemia	3 (2.3%)	3 (5.2%)	0.300 **
Folic Acid (Vit. B11)	5 (3.9%)	4 (7.0%)	0.363 **
Thiamine (Vit. B1)	6 (4.7%)	4 (7.0%)	0.517 **
Riboflavin (Vit. B2)	3 (2.3%)	5 (8.7%)	0.043 **
Niacin (Vit. B3)	4 (3.1%)	4 (7.0%)	0.229 **
Acid pantotenic (Vit. B5)	6 (4.7%)	5 (8.7%)	0.278 **
Pyridoxine (Vit. B6)	3 (2.3%)	4 (7.0%)	0.124 **
Biotina (Vit. B8)	4 (3.1%)	4 (7.0%)	0.229 **
Iodine	3 (2.3%)	2 (3.5%)	0.651 **
Zinco	3 (2.3%)	3 (5.2%)	0.300 **
Manganese	5 (3.9%)	6 (10.5%)	0.078 **
Calcium citrate	6 (4.7%)	7 (12.3%)	0.062 **

** Fisher’s exact test.

## Data Availability

The datasets used and/or analyzed during the current study are available from the corresponding author upon reasonable request.

## References

[1] Ghusn W., Hage K., Vierkant R.A. (2024). Type-2 diabetes mellitus remission prediction models after Roux-En-Y gastric bypass and sleeve gastrectomy based on disease severity scores. Diabetes Res. Clin. Pract..

[2] Fried M., Yumuk V., Oppert J.M., Scopinaro N., Torres A.J., Weiner R., Yashkov Y., Frühbeck G. (2013). European Association for the Study of Obesity, International Federation for the Surgery of Obesity—European Chapter: Interdisciplinary European guidelines on metabolic and bariatric surgery. Obes. Facts.

[3] Frühbeck G., Toplak H., Woodward E., Yumuk V., Maislos M., Oppert J.M. (2013). Executive Committee of the European Association for the Study of Obesity: Obesity: The gateway to ill health—An EASO position statement on a rising public health, clinical and scientific challenge in Europe. Obes. Facts.

[4] Affinati A.H., Esfandiari N.H., Oral E.A., Kraftson A.T. (2019). Bariatric Surgery in the Treatment of Type 2 Diabetes. Curr. Diabetes Rep..

[5] Wadden T.A., Chao A.M., Moore M. (2023). The Role of Lifestyle Modification with Second-Generation Anti-obesity Medications: Comparisons, Questions, and Clinical Opportunities. Curr. Obes. Rep..

[6] Barrea L., Vetrani C., Caprio M. (2023). Nutritional management of type 2 diabetes in subjects with obesity: An international guideline for clinical practice. Crit. Rev. Food Sci. Nutr..

[7] Hossain P., Kawar B., El Nahas M. (2007). Obesity and diabetes in the developing world—A growing challenge. N. Engl. J. Med..

[8] Ruze R., Liu T., Zou X., Song J., Chen Y., Xu R., Yin X., Xu Q. (2023). Obesity and type 2 diabetes mellitus: Connections in epidemiology, pathogenesis, and treatments. Front. Endocrinol..

[9] Arterburn D.E., Telem D.A., Kushner R.F., Courcoulas A.P. (2020). Benefits and Risks of Bariatric Surgery in Adults: A Review. JAMA.

[10] Cummings D.E., Rubino F. (2018). Metabolic surgery for the treatment of type 2 diabetes in obese individuals. Diabetologia.

[11] De Luca M., Zappa M.A., Zese M. (2022). Development of the Italian Clinical Practice Guidelines on Bariatric and Metabolic Surgery: Design and Methodological Aspects. Nutrients.

[12] Ghusn W., Ikemiya K., Al Annan K. (2023). Diabetes Mellitus Remission in Patients with BMI > 50 kg/m^2^ after Bariatric Surgeries: A Real-World Multi-Centered Study. Obes. Surg..

[13] Steven S., Hollingsworth K.G., Small P.K., Woodcock S.A., Pucci A., Aribasala B., Al-Mrabeh A., Batterham R.L., Taylor R. (2016). Calorie restriction and not glucagon-like peptide-1 explains the acute improvement in glucose control after gastric bypass in Type 2 diabetes. Diabetes Med..

[14] Balasubaramaniam V., Pouwels S. (2023). Remission of Type 2 Diabetes Mellitus (T2DM) after Sleeve Gastrectomy (SG), One-Anastomosis Gastric Bypass (OAGB), and Roux-en-Y Gastric Bypass (RYGB): A Systematic Review. Medicina.

[15] Chumakova-Orin M., Vanetta C., Moris D.P., Guerron A.D. (2021). Diabetes remission after bariatric surgery. World J. Diabetes.

[16] Abu-Abeid A., Lessing Y., Pencovich N., Dayan D., Klausner J.M., Abu-Abeid S. (2018). Diabetes resolution after one anastomosis gastric bypass. Surg. Obes. Relat. Dis..

[17] Peterli R., Wölnerhanssen B.K., Peters T., Vetter D., Kröll D., Borbély Y., Schultes B., Beglinger C., Drewe J., Schiesser M. (2018). Effect of Laparoscopic Sleeve Gastrectomy vs Laparoscopic Roux-en-Y Gastric Bypass on Weight Loss in Patients With Morbid Obesity: The SM-BOSS Randomized Clinical Trial. JAMA.

[18] von Elm E., Altman D.G., Egger M., Pocock S.J., Gøtzsche P.C., Vandenbroucke J.P. (2014). The Strengthening the Reporting of Observational Studies in Epidemiology (STROBE) Statement: Guidelines for reporting observational studies. Int. J. Surg..

[19] Pizza F., Lucido F.S., D’Antonio D. (2020). Biliopancreatic Limb Length in One Anastomosis Gastric Bypass: Which Is the Best?. Obes. Surg..

[20] Pizza F., D’antonio D., Lucido F.S., Tolone S., Del Genio G., Dell’isola C., Docimo L., Gambardella C. (2020). The Role of Ursodeoxycholic Acid (UDCA) in Cholelithiasis Management After One Anastomosis Gastric Bypass (OAGB) for Morbid Obesity: Results of a Monocentric Randomized Controlled Trial. Obes. Surg..

[21] Svanevik M., Lorentzen J., Borgeraas H. (2023). Patient-reported outcomes, weight loss, and remission of type 2 diabetes 3 years after gastric bypass and sleeve gastrectomy (Oseberg); a single-centre, randomised controlled trial. Lancet Diabetes Endocrinol..

[22] Mingrone G., Panunzi S., De Gaetano A., Guidone C., Iaconelli A., Capristo E., Chamseddine G., Bornstein S.R., Rubino F. (2021). Metabolic surgery versus conventional medical therapy in patients with type 2 diabetes: 10-year follow-up of an open-label, single-centre, randomised controlled trial. Lancet.

[23] Gagner M. (2019). For whom the bell tolls? It is time to retire the classic BPD (bilio-pancreatic diversion) operation. Surg. Obes. Relat. Dis..

[24] Angrisani L., Santonicola A., Iovino P., Formisano G., Buchwald H., Scopinaro N. (2015). Bariatric surgery worldwide 2013. Obes. Surg..

[25] Ahuja A., Tantia O., Goyal G., Chaudhuri T., Khanna S., Poddar A., Gupta S., Majumdar K. (2018). MGB-OAGB: Effect of Biliopancreatic Limb Length on Nutritional Deficiency, Weight Loss, and Comorbidity Resolution. Obes. Surg..

[26] De Luca M., Tie T., Ooi G., Higa K., Himpens J., Carbajo M.-A., Mahawar K., Shikora S., Brown W.A. (2018). Mini Gastric Bypass-One Anastomosis Gastric Bypass (MGB-OAGB)-IFSO Position Statement. Obes. Surg..

[27] Musella M., Apers J., Rheinwalt K., Ribeiro R., Manno E., Greco F., Čierny M., Milone M., Di Stefano C., Guler S. (2016). Efficacy of Bariatric Surgery in Type 2 Diabetes Mellitus Remission: The Role of Mini Gastric Bypass/One Anastomosis Gastric Bypass and Sleeve Gastrectomy at 1 Year of Follow-up. A European survey. Obes. Surg..

[28] Vrakopoulou G.Z., Theodoropoulos C., Kalles V., Zografos G., Almpanopoulos K. (2021). Type 2 diabetes mellitus status in obese patients following sleeve gastrectomy or one anastomosis gastric bypass. Sci. Rep..

[29] Ding Z., Jin L., Song Y., Feng C., Shen P., Li H. (2023). Comparison of single-anastomosis gastric bypass and sleeve gastrectomy on type 2 diabetes mellitus remission for obese patients: A meta-analysis of randomized controlled trials. Asian J. Surg..

[30] Shivakumar S., Tantia O., Goyal G., Chaudhuri T., Khanna S., Ahuja A., Poddar A., Majumdar K. (2018). LSG vs. MGB-OAGB3 year follow-up data: A randomised control trial. Obes. Surg..

[31] Kular K.S., Manchanda N., Rutledge R. (2014). Analysis of the five-year outcomes of sleeve gastrectomy and mini gastric bypass: A report from the indian sub-continent. Obes. Surg..

[32] Musella M., Berardi G., Vitiello A., Dayan D., Schiavone V., Franzese A., Abu-Abeid A. (2022). Vitamin D Deficiency in Patients with Morbid Obesity before and after Metabolic Bariatric Surgery. Nutrients.

[33] Castro M.-J., Jimenez J.-M., Carbajo M.-A., Lopez M., Cao M.-J., Garcia S., Ruiz-Tovar J. (2020). Long-Term Weight Loss Results, Remission of Comorbidities and Nutritional Deficiencies of Sleeve Gastrectomy (SG), Roux-En-Y Gastric Bypass (RYGB) and One-Anastomosis Gastric Bypass (OAGB) on Type 2 Diabetic (T2D) Patients. Int. J. Environ. Res. Public Health.

